# Association between Plasma 25-Hydroxyvitamin D, Ancestry and Aggressive Prostate Cancer among African Americans and European Americans in PCaP

**DOI:** 10.1371/journal.pone.0125151

**Published:** 2015-04-28

**Authors:** Susan E. Steck, Lenore Arab, Hongmei Zhang, Jeannette T. Bensen, Elizabeth T. H. Fontham, Candace S. Johnson, James L. Mohler, Gary J. Smith, Joseph L. Su, Donald L. Trump, Anna Woloszynska-Read

**Affiliations:** 1 Department of Epidemiology and Biostatistics, Center for Research in Nutrition and Health Disparities, Cancer Prevention and Control Program, Arnold School of Public Health, University of South Carolina, Columbia, South Carolina, United States of America; 2 David Geffen School of Medicine, University of California Los Angeles, Los Angeles, California, United States of America; 3 University of Memphis, Memphis, Tennessee, United States of America; 4 Department of Epidemiology, Gillings School of Global Public Health, Lineberger Comprehensive Cancer Center, University of North Carolina at Chapel Hill, Chapel Hill, North Carolina, United States of America; 5 School of Public Health, Louisiana State University Health Sciences Center, New Orleans, Louisiana, United States of America; 6 Roswell Park Cancer Institute, Buffalo, New York, United States of America; 7 Division of Cancer Control and Population Sciences, National Cancer Institute, Bethesda, Maryland, United States of America; Hormel Institute, University of Minnesota, UNITED STATES

## Abstract

**Background:**

African Americans (AAs) have lower circulating 25-hydroxyvitamin D3 [25(OH)D3] concentrations and higher prostate cancer (CaP) aggressiveness than other racial/ethnic groups. The purpose of the current study was to examine the relationship between plasma 25(OH)D3, African ancestry and CaP aggressiveness among AAs and European Americans (EAs).

**Methods:**

Plasma 25(OH)D3 was measured using LC-MS/MS (Liquid Chromatography Tandem Mass Spectrometry) in 537 AA and 663 EA newly-diagnosed CaP patients from the North Carolina-Louisiana Prostate Cancer Project (PCaP) classified as having either ‘high’ or ‘low’ aggressive disease based on clinical stage, Gleason grade and prostate specific antigen at diagnosis. Mean plasma 25(OH)D3 concentrations were compared by proportion of African ancestry. Logistic regression was used to calculate multivariable adjusted odds ratios (OR) and 95% confidence intervals (95%CI) for high aggressive CaP by tertile of plasma 25(OH)D3.

**Results:**

AAs with highest percent African ancestry (>95%) had the lowest mean plasma 25(OH)D3 concentrations. Overall, plasma 25(OH)D3 was associated positively with aggressiveness among AA men, an association that was modified by calcium intake (OR_T3vs.T1_: 2.23, 95%CI: 1.26–3.95 among men with low calcium intake, and OR_T3vs.T1_: 0.19, 95%CI: 0.05–0.70 among men with high calcium intake). Among EAs, the point estimates of the ORs were <1.0 for the upper tertiles with CIs that included the null.

**Conclusions:**

Among AAs, plasma 25(OH)D3 was associated positively with CaP aggressiveness among men with low calcium intake and inversely among men with high calcium intake. The clinical significance of circulating concentrations of 25(OH)D3 and interactions with calcium intake in the AA population warrants further study.

## Introduction

African Americans (AAs) are diagnosed with aggressive prostate cancer more often and have more than twice the prostate cancer mortality rates as European Americans (EAs) [1,2]. Vitamin D has been hypothesized to play a role in explaining some of the racial disparities in cancer mortality for various cancer types [3–5]. Mean circulating 25-hydroxyvitamin D3 [25(OH)D3, the metabolite measured to assess vitamin D status clinically] is lower among AAs than EAs [5–9]. Vitamin D in humans is derived from cutaneous exposure to ultraviolet (UV)-B rays from sunlight and the conversion of 7-dehydrocholesterol to pre-vitamin D3 with subsequent hepatic and renal conversion of D3 to the most active metabolite 1,25-dihydroxyvitamin D3 [1,25(OH)_2_D3] [10]. Melanin blocks UV-B, and individuals with darker skin pigmentation require longer time in the sun to produce equivalent amounts of 25(OH)D3 than individuals with less pigmented skin [11]. Dietary and supplemental intake contribute proportionally much less to vitamin D status. Intake of vitamin D is lower in AAs than in EAs which may reflect^{16^ low intake of milk products among AAs due to a higher prevalence of lactose intolerance [12–14]. African ancestry is inversely associated with serum 25(OH)D3 concentrations [15] and somewhat correlated with skin pigmentation [16,17]. The proportion of African ancestry may be related to genetic differences that affect metabolism, activity or storage of vitamin D.

The epidemiological evidence for an association between circulating 25(OH)D3 and prostate cancer risk has been reviewed [18–20], though few studies have included a large enough sample of AAs to examine intra-racial associations with circulating concentrations of vitamin D [21,22]. The majority of studies have found either null or inverse associations [23–33], but several studies have shown increased risk of prostate cancer at the highest concentrations of 25(OH)D3 [34–38]. Other studies reported a U- or J-shaped association between 25(OH)D3 and prostate cancer risk [39,40] or overall mortality [41,42]. A recent meta-analysis reported inverse associations with overall cancer mortality for supplementation trials of vitamin D3 but not D2, and no substantial association between circulating 25(OH)D and prostate cancer-specific death [43]. Calcium and vitamin D levels are regulated homeostatically. An interaction between calcium intake and vitamin D status in relation to prostate cancer has been suggested, but rarely reported [34,38].

Given the higher incidence of aggressive prostate cancer among AAs, the conflicting evidence for an association between 25(OH)D3 and prostate cancer, and the underrepresentation of AA in previously published research, the goals of this study were to examine the association between plasma 25(OH)D3 concentrations and prostate cancer aggressiveness in a large, population-based, case-only study of AA and EA men newly diagnosed with prostate cancer, to examine potential interactions with calcium intake, and to examine if African ancestry as measured by ancestry informative markers could shed more light on any such race-specific relationships.

## Materials and Methods

### Study Population

This study utilizes data from a subset of research subjects in the North Carolina-Louisiana Prostate Cancer Project (PCaP), a population-based, case-only study designed to address racial disparities in prostate cancer. A subset of PCaP participants (n = 1200) who had agreed to future use of their biologic specimens for research were selected *a priori* for inclusion in this study due to resource constraints. The study set consisted of all research subjects diagnosed with high aggressive prostate cancer (n = 302, see aggressiveness definition in Outcomes Assessment section), 112 research subjects diagnosed with Gleason score = 4+3 (all other intermediate aggressive cancer research subjects were excluded), and a random subset (n = 786) of research subjects diagnosed with low aggressive cancer (a random subset was selected because there were many more low aggressive cancer cases than needed for analyses). This research subject selection strategy was implemented prior to any 25(OH)D3 lab measurements or data analyses. PCaP methods have been described in detail [44]. Briefly, residents of the North Carolina (NC) and Louisiana (LA) study areas between the ages of 40–79 years old with an initial diagnosis of histologically-confirmed adenocarcinoma of the prostate between July 2004 and July 2009 were eligible. The sampling frame was weighted to include all eligible AAs, and EAs were under-sampled randomly using randomized recruitment (36). The PCaP protocol was approved by the institutional review boards at all participating sites, which included the two patient recruitment sites [the University of North Carolina at Chapel Hill (UNC) and Louisiana State University Health Sciences Center (LSUHSC)], and the Department of Defense Prostate Cancer Research Program. Prior to their participation in PCaP, all men signed an informed consent and provided signed release for medical records and tumor specimens.

### Data Collection

Research subjects were visited in their home by a trained registered nurse who conducted a structured interview, performed anthropometric measurements, and collected biospecimens. The majority of visits were completed on the average within four months of diagnosis. Structured questionnaires were used to collect information about lifestyle factors, family history of prostate cancer, cancer screening history, and prescribed and over-the-counter medications used in the prior two weeks, which included non-steroidal anti-inflammatory drugs (NSAIDs), vitamins and supplements. Men were asked to report usual dietary intake in the year prior to diagnosis using the National Cancer Institute Diet History Questionnaire (DHQ) modified to capture foods common to the geographic areas (e.g., Cajun and creole foods). The modified DHQ inquired about frequency of intake and usual portion size for 124 food items, and food preparation methods. Questionnaire responses were linked to the DHQ Nutrient Database through the Diet*Calc software, and intakes of macronutrients, micronutrients, and minerals, including calcium, were computed. After the in-home visit, medical records and tumor tissue samples were collected for each research subject who provided authorization for release.

### Vitamin D Assessment

During in-home visits, study nurses collected 6.5 ml of fasting venous blood into lavender top (EDTA) tubes which were wrapped in foil and transported on ice at 4°C to the Blood and Tissue Procurement Core Laboratory at LSU or the BioSpecimen Processing Facility at UNC. The majority of PCaP blood samples were processed to serum, plasma and DNA on the same or the following day and aliquoted and stored at -80°C. Plasma concentrations of 25(OH)D3 were determined using LC-MS/MS at Heartland Assays, Inc. PCaP plasma samples were stored at -80°C for up to eight years prior to measurement; concentrations of 25(OH)D3 in stored samples has been reported to be quite stable even at -20°C for up to ten years [45].

### Ancestry Informative Markers

Ancestry informative markers were genotyped and ancestry estimates (i.e., proportion African descent) were determined as described [46]. Allele frequencies were estimated using maximum likelihood methods. Individual ancestry proportions for self-reporting AA and EA research subjects were estimated using a Bayesian Markov Chain Monte Carlo (MCMC) clustering algorithm implemented in STRUCTURE 2.3.1. Publicly available genotypes were included from YRI, CEU and ASI ancestral populations in the STRUCTURE procedure.

### Outcome Assessment

Extensive medical record abstraction was performed in PCaP and data on Gleason grade, clinical stage, and prostate specific antigen (PSA) at diagnosis were used to classify research subjects into three categories of aggressiveness as follows [44]: High aggressive cases: Gleason sum ≥ 8, or PSA >20 ng/ml at diagnosis, or Gleason sum = 7 AND stage T3-T4; low aggressive cases: Gleason sum <7 AND diagnosed at stage T1-T2 AND PSA <10 ng/ml at diagnosis; intermediate aggressive cases: all other cases. For the current study, all PCaP research subjects diagnosed with high aggressive cancer and intermediate aggressive cancer research subjects who had Gleason sum = 7 with primary Gleason pattern 4 were combined (this group is referred to as ‘high aggressive’). The high aggressive group was compared to a random sample of low aggressive cases with Gleason sum <7, stage T1-T2, and PSA<9 ng/ml (as described in Study Population above).

### Statistical analysis

Research subjects were excluded who had incomplete data on confounders or effect modifiers (40 total exclusions). The final study sample consisted of 519 AA and 641 EA men. Descriptive analyses included calculating means and standard deviations for continuous variables and frequencies and percentages for categorical variables, which included frequencies by predetermined categories of 25(OH)D3 based on previous literature (<20ng/ml, 20–30ng/ml, and ≥30ng/ml) and by tertiles based on the 25(OH)D3 race-specific (self-reported race) distributions in PCaP. Means and standard deviations of plasma 25(OH)D3 were calculated for EAs and AAs, and by African ancestry, categorized as >95%, 85–95%, <85% based on ancestry informative markers to be comparable to previous literature [15]. Crude and multivariable logistic regressions were used to examine the relationships between plasma 25(OH)D3 concentrations and prostate cancer aggressiveness by comparing high aggressive research subjects to low aggressive research subjects. The distributions of plasma 25(OH)D3 were different between AAs and EAs, so all analyses were stratified by race and plasma 25(OH)D3 concentrations were grouped into tertiles (T) with race-specific cut-off points based on the distribution among low aggressive prostate cancer cases; for AAs: T1<13.30, 13.30 ≤ T2 < 18.90, T3 ≥18.90ng/ml; and for EAs: T1<21.14, 21.14 ≤ T2 < 26.67, T3 ≥26.67 ng/ml. The lowest race-specific tertile was used as the referent in race-stratified analyses. Confounding was assessed using the 10% rule. Final multivariable logistic regression models adjusted for age (years, continuous); African ancestry (%, continuous); body mass index (BMI; kg/m^2^, continuous); education (less than 8^th^ grade or some high school; high school graduate or vocational/technical school; some college or college graduate; some graduate training or graduate/professional degree); PSA screening history (0, 1–7, >7 screenings); smoking (non-smoker, former smoker, and current smoker); alcohol intake (g/day, continuous); NSAID use (yes/no); study site (NC, LA); season of blood draw (winter, spring, summer, and fall); physical activity (MET-hrs/wk, continuous); and total energy intake (kcal/day, continuous). Based on 10% rule, marital status and family history of first degree relative with prostate cancer were not confounders and were not included in the final model. Stratified analyses were performed to explore possible effect modifying role of age (< 65, ≥ 65 years), BMI (< 30, ≥30 kg/m^2^), study site (NC, LA), and dietary calcium intake (< 1200, ≥ 1200 mg/d) in the relationships between plasma 25(OH)D3 concentrations and prostate cancer aggressiveness, and interactions were tested by including an interaction term in fully adjusted race-stratified models. Sensitivity analyses were performed to examine effects of recent weight change (measured weight at time of interview minus self-report weight one year prior to diagnosis), number of days until processing of blood sample (0–1 day compared to ≥2 days; 10% were processed ≥2 days), or current use of vitamin D supplements at time of blood draw. In addition, research subjects with Gleason score = 4+3 (included in the high risk group in this study, but classified as intermediate aggressive by PCaP) were excluded in sensitivity analyses to restrict the high aggressive group to those subjects with Gleason score of 8 or greater in order to evaluate whether including less aggressive cancers in the high aggressive group may have biased the associations observed. SAS version 9.3 (SAS Institute, Cary, NC) was used for all analyses, and statistical significance was evaluated at p<0.05 (two-tailed).

## Results

Data from 306 AAs and 456 EAs with low aggressive prostate cancer, and 213 AAs and 185 EAs with high aggressive prostate cancer were included in the analyses. The majority of PCaP research subjects had no family history of prostate cancer, were former smokers, were married or living with a partner, and were not taking vitamin D supplements at the time of interview (Table 1). Almost half of interviews occurred during the Fall (September 21 to December 20). Average age of research subjects was 63 years and average BMI was 29.3 kg/m^2^. Low and high aggressive cases were distributed similarly at the NC and LA study sites

**Table 1:** Descriptive statistics by prostate cancer aggressiveness and race.

Characteristics	Low Aggressive				High Aggressive			
	AA		EA		AA		EA	
**Continuous variables**	**Mean**	**SD**	**Mean**	**SD**	**Mean**	**SD**	**Mean**	**SD**
Age, yrs	61	8	63	7	62	8	66	8
Body mass index, kg/m^2^	28.8	5.3	29.0	4.8	29.4	6.6	29.8	5.0
Total energy intake, kcal/day	2638.2	1334.3	2355.0	1155.1	3033.0	1607.2	2504.2	1269.6
Alcohol intake, g/day	17.3	45.1	14.3	43.2	20.0	63.0	19.3	49.4
Total vitamin D intake, mcg/d	6.3	7.1	7.7	8.0	7.2	18.9	8.8	9.3
Calcium intake, mg/d	804.3	471.4	923.9	488.4	929.1	516.4	1020.4	585.3
Physical activity, MET-hours/week	3.6	5.1	4.3	5.5	3.3	5.0	4.2	5.6
Plasma 25(OH)D3, ng/ml	17.1	7.4	24.5	8.6	18.4	7.7	25.0	12.0
**Categorical variables**	**n**	**%**	**n**	**%**	**n**	**%**	**n**	**%**
**Study Site**								
NC	151	49.4	223	51.1	101	47.4	87	47.0
LA	155	50.6	233	48.9	112	52.6	98	53.0
**Family History of Prostate Cancer**								
No affected 1^st^ degree relative	196	69.5	323	72.9	146	73.4	143	75.7
At least 1 affected 1^st^ degree relative	86	30.5	120	27.1	53	26.6	33	18.7
**Education**								
Grad/professional degree	24	7.8	98	21.5	7	3.3	41	22.2
Some college or college graduate	95	31.1	189	41.5	60	28.2	77	41.6
High school grad or voc/tech school	107	35.0	136	29.8	68	31.9	41	22.2
Less than high school education	80	26.1	33	7.2	78	36.6	26	14.0
**PSA Screening History**								
0 screenings	96	31.4	69	15.1	115	54.0	33	17.8
1–7 screenings	137	44.8	206	45.2	60	28.2	79	42.7
> 7 screenings	73	23.8	181	39.7	38	17.8	73	39.5
**Smoking Status**								
Current smokers	114	37.3	179	39.3	44	20.7	69	37.3
Former smokers	131	42.8	240	52.6	117	54.9	97	52.4
Non-smokers	61	19.9	37	8.1	52	24.4	19	10.3
**NSAID Use**								
No	147	48.0	150	31.6	82	38.5	65	35.1
Yes	159	52.0	323	68.4	131	61.5	120	64.9
**Taking dietary supplement containing vitamin D at the time of the interview**								
No	253	81.9	312	70.0	173	79.4	118	63.1
Yes	56	18.1	144	30.0	45	20.6	69	26.9
**Season**								
Winter (21Dec–22Mar)	55	18.0	81	17.7	26	12.2	31	16.8
Spring (21Mar–20Jun)	54	17.6	88	19.3	46	21.6	35	18.9
Summer (21Jun–20Sep)	53	17.3	71	15.6	34	16.0	32	17.3
Fall (21Sep–20Dec)	144	47.1	216	47.4	107	50.2	87	47.0
**African Ancestry (among African Americans)**								
High (>0.95)	197	64.4	NA	NA	132	62.0	NA	NA
Medium (0.85–<0.95)	49	16.0	NA	NA	35	16.4	NA	NA
Low (<0.85)	60	19.6	NA	NA	46	21.6	NA	NA
**Marital Status**								
Single/separated/divorced/widowed	95	31.1	59	12.9	74	34.7	39	21.1
Married/living with partner	211	68.9	397	87.1	139	65.3	146	78.9

**Fig 1:**
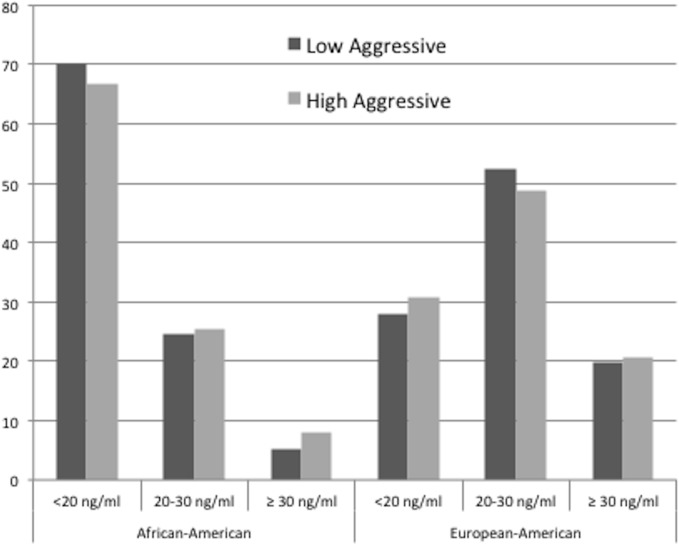
Distribution of plasma 25(OH)D3 by prostate cancer aggressiveness and race.

The majority of AAs (67% of high aggressive and 70% of low aggressive cases) had 25(OH)D3 concentrations below 20ng/ml, compared to approximately 30% of EAs (31% of high aggressive and 28% of low aggressive) (Fig 1). Research subjects with higher proportions of African ancestry, as indicated by ancestry informative genetic markers, had lower mean concentrations of plasma 25(OH)D3 (17.0 ± 7.2, 18.4 ± 8.3, and 19.1 ± 7.6 ng/ml for AAs with African ancestry of >95%, 85–95%, and <85%, respectively), whereas the mean concentration of 25(OH)D3 among self declared EA men (all with <40% African ancestry) was 24.6 ± 9.7 ng/ml.

Among AAs, higher odds of high aggressive prostate cancer were observed for research subjects in the second and third tertiles of 25(OH)D3 concentration compared to the first, after adjustment for potential confounders (OR_T2 vs T1_: 1.80, 95%CI: 1.10–2.96 and OR_T3 vs. T1_: 1.46, 95%CI: 0.89–2.39, Table 2). In contrast, the point estimates of the ORs for EAs were less than 1.0 but not statistically significantly different from 1.0 (OR_T2 vs T1_: 0.92, 95%CI: 0.59–1.43 and OR_T3 vs. T1_: 0.92, 95%CI: 0.58–1.44). The associations between 25(OH)D3 and prostate cancer aggressiveness were not modified by age, BMI, or study site (Table 3). However, dietary calcium intake appeared to modify the association among AAs (P_interaction_ = 0.001). High aggressive prostate cancer was associated with higher 25(OH)D3 concentrations among men with low calcium intake (<1200mg/d; OR_T3 vs. T1_: 2.23, 95%CI: 1.26–3.95). A decreased odds of high aggressive prostate cancer for higher 25(OH)D3 was observed among men with higher calcium intake (≥ 1200 mg/d; OR_T3 vs. T1_: 0.19, 95%CI: 0.05–0.70). The interaction p-value for EAs was not significant (p = 0.99), though the effect estimates were in a similar direction as those of AAs in men with higher calcium intake.

**Table 2:** Age- and multivariable-adjusted odds ratios and 95% confidence intervals for high aggressive prostate cancer by tertiles of plasma 25(OH)D3.

	25(OH)D3 tertiles, ng/ml	n (high aggressive/ low aggressive)	Age-adjusted odds ratio	Age-adjusted 95% CI	Fully adjusted^a^ odds ratio	Fully adjusted^a^ 95% CI	Fully adjusted^a^ trend test p-value
**African Americans**	< 13.30	56/102	1.00	referent	1.00	referent	
	13.30 ≤ T2 < 18.90	76/100	1.40	0.89, 2.18	1.80	1.10, 2.96	
	≥ 18.90	81/104	1.36	0.88, 2.11	1.46	0.89, 2.39	0.27
**European Americans**	< 21.14	67/155	1.00	referent	1.00	referent	
	21.14 ≤ T2 < 26.67	58/150	0.90	0.59, 1.37	0.92	0.59, 1.43	
	≥ 26.67	60/151	0.88	0.58, 1.34	0.92	0.58, 1.44	0.71

^a^Adjusted for age, African ancestry, BMI, total energy intake, alcohol intake, physical activity, smoking status, educational status, PSA screening history, study site, NSAIDs use, and season of blood draw

**Table 3:** Association between measured concentrations of plasma 25(OH)D3 and prostate cancer aggressiveness stratified by race and selected demographic, clinical and dietary characteristics.

Race	Characteristic	Tertiles^a^			P_interaction_
		1	2	3	
**Age, years**					
**African Americans**	< 65				
	High aggressive/ low aggressive, n	35/65	47/75	44/67	
	OR (95% CI)^b^	1.00 (Ref.)	1.70 (0.90–3.19)	1.52 (0.80–2.86)	
	≥ 65				
	High aggressive/ low aggressive, n	21/37	29/25	37/37	
	OR (95% CI) ^b^	1.00 (Ref.)	2.42 (0.97–6.06)	1.72 (0.70–4.22)	0.82
**European Americans**	< 65				
	High aggressive/ low aggressive, n	28/91	23/87	23/83	
	OR (95% CI)^b^	1.00 (Ref.)	0.85 (0.44–1.65)	0.97 (0.49–1.93)	
	≥ 65				
	High aggressive/ low aggressive, n	39/64	35/63	37/68	
	OR (95% CI)^b^	1.00 (Ref.)	0.89 (0.49–1.64)	0.83 (0.44–1.55)	0.91
**BMI (kg/m** ^**2**^ **)**					
**African Americans**	< 30				
	High aggressive/ low aggressive, n	33/65	43/57	57/74	
	OR (95% CI) ^c^	1.00 (Ref.)	1.87 (0.96–3.65)	1.52 (0.80–2.88)	
	≥ 30				
	High aggressive/ low aggressive, n	23/37	33/43	24/30	
	OR (95% CI) ^c^	1.00 (Ref.)	1.71 (0.77–3.84)	1.05 (0.43–2.56)	0.78
**European Americans**	< 30				
	High aggressive/ low aggressive, n	28/93	37/104	39/103	
	OR (95% CI) ^c^	1.00 (Ref.)	1.19 (0.64–2.19)	1.20 (0.65–2.22)	
	≥ 30				
	High aggressive/ low aggressive, n	39/62	21/46	21/48	
	OR (95% CI) ^c^	1.00 (Ref.)	0.77 (0.38–1.58)	0.65 (0.31–1.36)	0.25
**Study Site**					
**African Americans**	**LA**				
	High aggressive/ low aggressive, n	22/46	40/49	50/60	
	OR (95% CI) ^d^	1.00 (Ref.)	2.18 (1.00–4.75)	1.77 (0.83–3.78)	
	**NC**				
	High aggressive/ low aggressive, n	34/56	36/51	31/44	
	OR (95% CI) ^d^	1.00 (Ref.)	1.58 (0.80–3.12)	1.33 (0.66–2.68)	0.78
				
**European Americans**	**LA**				
	High aggressive/ low aggressive, n	35/71	28/82	35/80	
	OR (95% CI) ^d^	1.0 (Ref.)	0.67 (0.35–1.27)	0.81 (0.43–1.54)	
	**NC**				
	High aggressive/ low aggressive, n	32/84	30/68	25/71	
	OR (95% CI) ^d^	1.00 (Ref.)	1.16 (0.60–2.21)	1.02 (0.51–2.03)	0.57
**Dietary Calcium Intake, mg/d**					
**African Americans**	**<**1200				
	High aggressive/ low aggressive, n	39/92	59/87	63/75	
	OR (95% CI) ^e^	1.00 (Ref.)	2.07 (1.17–3.66)	2.23 (1.26–3.95)	
	≥ 1200				
	High aggressive/ low aggressive, n	17/10	17/13	18/29	
	OR (95% CI) ^e^	1.00 (Ref.)	1.06 (0.28–3.99)	0.19 (0.05–0.70)	0.001
**European Americans**	< 1200				
	High aggressive/ low aggressive, n	51/126	42/117	38/108	
	OR (95% CI) ^e^	1.00 (Ref.)	0.97 (0.58–1.64)	0.97 (0.56–1.69)	
	≥ 1200				
	High aggressive/ low aggressive, n	16/29	16/33	22/43	
	OR (95% CI) ^e^	1.0 (Ref.)	0.77 (0.29–2.04)	0.87 (0.35–2.17)	0.99

^a^ Cutpoints for tertiles of 25(OH)D3 for African Americans: T1<13.30, 13.30 ≤ T2 < 18.90, T3 ≥18.90 ng/ml; for European Americans: T1<21.14, 21.14 ≤ T2 < 26.67, T3 ≥26.67 ng/ml

^b^ Adjusted for African ancestry, BMI, total energy intake, alcohol intake, physical activity, smoking status, educational status, PSA screening history, study site, NSAIDs use, and season of blood draw

^c^Adjusted for age, African ancestry, total energy intake, alcohol intake, physical activity, smoking status, educational status, PSA screening history, study site, NSAIDs use, and season of blood draw

^d^Adjusted for age, African ancestry, BMI, total energy intake, alcohol intake, physical activity, smoking status, educational status, PSA screening history, NSAIDs use, and season of blood draw

^e^Adjusted for age, African ancestry, BMI, total energy intake, alcohol intake, physical activity, smoking status, educational status, PSA screening history, study site, NSAIDs use, and season of blood draw

In sensitivity analyses, a variable for recent weight change [measured weight in kilograms (kg) at time of interview minus self-reported weight one year prior to diagnosis in kg converted from pounds (lbs)] was included in the models, and there was no substantial change (i.e., >10% change in effect estimates) in the results. Adjusting for number of days to processing the blood sample (blood samples from 126 men were processed two days after collection and from one man five days after collection, whereas all others were processed on the same or next day after collection, with the majority, n = 650 processed on the same day) did not affect the results, neither did adjusting for vitamin D supplement use at the time of the interview. The OR for AA men in the highest tertile of 25(OH)D3 compared to the lowest strengthened to 1.76 (95%CI: 1.02–3.05) after excluding those research subjects with ≥two days between collection and processing. Excluding men who reported taking a vitamin D supplement at the time of interview (19% of AAs and 32% of EAs) or excluding men with Gleason score = 4+3 (originally classified by PCaP as intermediate aggressive) did not change the results of these analyses.

## Discussion

This population-based case-only study of AA and EA men with incident prostate cancer confirms the inverse relationship between African ancestry and circulating vitamin D concentrations. A novel finding is that higher total plasma 25(OH)D3 concentrations were associated with increased odds of high aggressive prostate cancer among AAs. This contrasts with the findings among EAs where point estimates suggested a modest protective effect of higher concentrations which was not statistically significant after adjustment for potential confounders. Associations were not modified by age, BMI, or study site. However, a significant interaction between self-reported calcium intake and measured plasma 25(OH)D3 concentration was observed among AAs. The positive association between 25(OH)D3 concentration and high aggressive prostate cancer was observed in men with lower calcium intake (<1200mg/d), whereas higher 25(OH)D3 was associated with reduced odds of high aggressive prostate cancer among men with higher calcium intake (≥1200mg/d).

Similar to previous studies [15,47], mean concentrations of circulating 25(OH)D3 varied by proportion of African ancestry; the lowest mean concentration occurred in men with >95% African ancestry. The distribution of plasma 25(OH)D3 concentration differed between AAs and EAs; race-specific cutpoints had to be used to ensure ample numbers of research subjects in each category and to produce meaningful results when stratified by race. Approximately 70% of AA prostate cancer cases but only approximately 30% of EA cases had 25(OH)D3 concentrations <20ng/ml. While the distribution of African ancestry was not substantially different between NC and LA in this PCaP subset, a higher percentage of AAs from NC versus LA had 25(OH)D3 concentrations <20 ng/ml (74% versus 64%, respectively), which may reflect variations in sunlight exposure and diet between research subjects from the two states.

The findings of increased odds of high aggressive prostate cancer at higher plasma 25(OH)D3 concentrations among AAs is counter to the original hypothesis that higher vitamin D may be protective against aggressive prostate cancer and contrasted to some epidemiological studies supporting the original hypothesis [21,22,30,48,49]. However, several nested case-control studies have reported increased risk of aggressive prostate cancer [34,38] or total prostate cancer [35,37] for men with higher concentrations of 25(OH)D3. The majority of these studies were in European or EA populations (or did not report results separately by race), with 25(OH)D3 concentrations greater than approximately 32ng/ml associated with increased risk. These concentrations are much higher than those observed in PCaP AA research subjects at higher odds of aggressive prostate cancer (second tertile cutpoint >13.3 ng/ml). The differences in study populations, choice of control groups, and distribution of plasma 25(OH)D3 concentrations limit ability to compare results across studies. Only 5% of AAs had 25(OH)D3 concentrations above 32ng/ml in PCaP. Thus, it is unclear what the effect of higher concentrations of 25(OH)D3 would be in this population.

In contrast with our findings, Murphy et al. reported that AA (and EA) men with low serum 25(OH)D concentrations (<12 ng/ml compared to >12ng/ml) had higher odds of higher Gleason grade and higher tumor stage in a cross-sectional study performed in Chicago, IL [21]. Kristal et al. recently reported reduced risk of Gleason 7–10 prostate cancer among AAs in the upper three quintiles of serum 25(OH)D compared to the lower two quintiles in the Selenium and Vitamin E Cancer Prevention Trial [22]. In a recent randomized, placebo-controlled clinical trial, varying doses of vitamin D supplementation had no effect on PSA levels in healthy AA men without prostate cancer [50]. The current study results, along with these recent reports underscore the need for more research on vitamin D and prostate health among AAs who have been underrepresented in previous research studies.

Experimental models support a role of vitamin D in halting prostate cancer progression which contrasts with inconsistent findings in the epidemiologic literature. Vitamin D has been reported to inhibit proliferation in prostate cancer cell lines and inhibit invasion and metastases in some prostate cancer animal models, such as the Dunning rat model [51–55]. The balance of 25(OH)D3 concentrations and 24-hydroxylation that inactivates 1,25(OH)_2_D3 at the target tissue may affect the response to circulating 25(OH)D3 [39]. Clinical cutpoints for assessing vitamin D status remain unstandardized, but the Institute of Medicine recommended a 25(OH)D3 cutpoint of >20ng/ml as sufficient for skeletal health outcomes [56]. Recent research calls into question the uniform application of cutpoints across all racial groups [57]. The majority of 25(OH)D3 is bound to vitamin D binding protein in circulation [58]. Genotypes of the gene encoding the vitamin D binding protein (*GC*) are distributed differentially across races; AAs have a higher prevalence of genotypes associated with low levels of vitamin D binding protein [59–62]. A recent study reported that AAs and EAs had similar concentrations of free 25(OH)D [considered to be more bioavailable than bound 25(OH)D], after *GC* genotypes were accounted for, which may explain the paradox of higher bone mineral density among AAs than EAs even in the presence of lower total 25(OH)D concentrations [57]. Levels of vitamin D binding protein were not available in the current study, though genotyping of genes involved in vitamin D metabolism and activity (including the vitamin D binding protein, *GC*) are underway.

Plasma samples were collected after diagnosis, so reverse causality is a possible explanation for the findings; disease status may have affected plasma 25(OH)D3 concentrations. Vitamin D is fat-soluble and stored in adipose tissue. One possible explanation for the unexpected findings may be that men with high aggressive disease were more likely to have lost weight recently than those with low aggressive disease, which would release 25(OH)D3 into circulation from adipose tissue and increase plasma concentrations of 25(OH)D3. However, adjustment for recent weight change in the year prior to diagnosis did not change the association between 25(OH)D3 concentrations and aggressiveness. Systematic differences in the way blood samples were collected and processed were investigated, but adjustment for time to blood processing and exclusion of those research subjects with two or five days before processing also did not substantially affect results. Another potential explanation for the unexpected finding was the possibility that research subjects diagnosed with prostate cancer may have started supplementation with vitamin D after diagnosis but before the interview, and that this would have been done differentially by AA research subjects with high versus low aggressive prostate cancer either due to physician recommendation or of their own volition. Nurse interviewers conducted an inventory of all medications and supplements used by research subjects at the time of the home visit. A slightly higher percentage of AA men with high versus low aggressive prostate cancer reported taking vitamin D supplements (21% versus 18%, respectively), but adjustment for recent use of vitamin D supplements or exclusion of those subjects reporting vitamin D supplement use did not change the results.

The current study found an interaction between calcium intake and plasma 25(OH)D3 concentrations among AAs. The positive association between 25(OH)D3 and high aggressive prostate cancer was observed in men with low calcium intakes (below the Recommended Daily Allowance of 1200 mg/d for men 71 years and older), whereas reduced odds of high aggressive prostate cancer for high concentrations of 25(OH)D3 was observed among AA men with higher calcium intakes. These results contrast with the Albanes et al. report of the Alpha-tocopherol, Beta-carotene Cancer Prevention Study in Finnish men that increased risk of prostate cancer for higher concentrations of serum 25(OH)D was more prominent among men with higher intakes of calcium (≥1338 mg/d) [38]. The Prostate, Lung, Colorectal and Ovarian Cancer Screening Trial reported no difference in effect of 25(OH)D3 on prostate cancer across all levels of calcium intake [34]. It is not clear if the finding in PCaP of an interaction with calcium represents a true biologic interaction perhaps related to genetic predisposition to lactose intolerance or may be due to chance given the small number of research subjects with calcium intake ≥1200 mg/d. Further research should evaluate the interaction between calcium and vitamin D in prostate cancer.

Strengths of the study include the use of rapid case ascertainment in identification of a population-based sample of a large number of clinically and epidemiologically well-characterized AA and EA men with incident prostate cancer from two study sites in the United States. Additionally, all men were genotyped for ancestry informative markers and thus proportion of African ancestry was available and used to reduce confounding by population stratification. Sunlight exposure produces as much as 90% of the vitamin D requirement for humans [63]. Plasma concentrations as the exposure variable provide a better indicator of vitamin D status than dietary intake or supplement data alone. Plasma 25(OH)D3 concentrations can vary by season, so season of blood collection was controlled for in the analyses. Nutrient biomarkers, such as plasma 25(OH)D3, provide more objective markers of exposure than participant recall of diet and supplement use. However, one measurement of plasma reflects only recent exposure, though 25(OH)D3 concentrations and skin color, the major determinant of 25(OH)D3, are relatively stable over time [28,64]. Other limitations of this study include those inherent to any observational epidemiological investigation, such as non-participation and the possibility of selection bias or recall bias for self-reported covariates.

In conclusion, an interaction between plasma concentrations of 25(OH)D3 and calcium intake was observed in relation to odds of high aggressive prostate cancer among AAs but not EAs. The study results add to the growing literature [22,40] suggesting that the relationship between 25(OH)D3 and disease is complex particularly in the context of cancer-related racial disparities. Recommendations for universal cutpoints of vitamin D status and increasing 25(OH)D3 concentrations in all populations may not be ideal. Other factors may need to be considered which include calcium intake, genetic ancestry, background variation, and concentrations of bioavailable vitamin D.
